# Role of simultaneous bilateral transforaminal epidural steroid injections in patients with prior lumbar fusions or laminectomies: A retrospective case series

**DOI:** 10.1016/j.inpm.2022.100066

**Published:** 2022-01-26

**Authors:** Francine Zeng, Scott Mallozzi, Isaac Moss, Mark Cote, Durgadas Sakalkale

**Affiliations:** a University of Connecticut School of Medicine, Farmington, CT, USA; b Department of Orthopaedic Surgery, University of Connecticut Health Center, Farmington, CT, USA

**Keywords:** Epidural, Transforaminal, Injections, Failed back surgery syndrome

## Abstract

**Objective:**

The goal of this study is to assess the clinical effectiveness and prognostic potential of simultaneous bilateral lumbar transforaminal epidural steroid injections (TFESIs) in patients with bilateral radicular back pain with previous history of lumbar laminectomy and/or fusion surgery.

**Design:**

Retrospective case series.

**Setting:**

Single physiatrist in an academic setting.

**Subjects:**

23 patients with previous lumbar surgery who received bilateral TFESIs.

**Methods:**

Cumulative bilateral radicular back pain was assessed with a pain numerical rating scale (NRS, 0–10) prior to receiving bilateral TFESI and at minimum 2 weeks follow-up. Responders included patients who experienced any NRS pain reduction post-procedure and non-responders were patients who reported no change in pain. A minimal clinically important difference (MCID) was defined as NRS change ≥2.0 to identify the proportion of responders who experienced a clinically significant reduction in pain. Other outcome measures included subsequent repeat bilateral TFESI, operations at the level of injections, and operative outcomes of these patients.

**Results:**

There was a statistically significant (P ​< ​0.0001) reduction of 2.2 in mean NRS at average 3.7 weeks follow-up. With the MCID defined as NRS pain reduction ≥2, 13 of 16 responders (56%; CI 36.8–74.4%) achieved a clinically significant reduction in pain. Nine patients (39.1%) went on to receive repeat bilateral TFESIs and 9 patients (39.1%) underwent surgical interventions involving the same spinal level as the injections. Eight of the 9 patients who underwent repeat bilateral TFESIs met follow-up criteria and each responded to repeat injections with an average NRS pain reduction of 2.2. Of the 9 surgical patients, 5 responded to the previous injections and each reported improvements in pain and function after their operations (PPV ​= ​100%). Of the 4 surgical patients who were non-responders to the injections, 2 reported improvements in pain and function post-operatively and the remaining 2 reported no change or worsening outcomes (NPV ​= ​50%).

**Conclusion:**

This study suggests bilateral TFESIs are clinically effective in short-term management of bilateral radicular back pain in patients with previous lumbar surgery, and they reveal potential prognostic information for subsequent surgical intervention.

## 1 Introduction

Epidural steroid injections have been utilized to treat lower back and radicular pain symptoms since the 1950s [1,2]. Over the years, epidural injections have demonstrated efficacy in managing radicular back pain [2], providing pain relief and improving function, decreasing opioid dependence, and reducing the need for surgical intervention [2]. Transforaminal epidural steroid injections (TFESI) represent a relatively new approach for radicular pain symptoms secondary to disc herniation or stenosis [2]. They can be performed both unilaterally and bilaterally, but most published studies have utilized unilateral injections. There have been several studies demonstrating their efficacy in both short-term and long-term treatment of radicular pain [2,3], with their targets being the anterolateral epidural space and dorsal root ganglion [3,4]. Examples of advantages of the transforaminal approach are its specificity with respect to specific nerve root symptoms, as well as requiring a smaller injectate volume in order to provide symptom relief. However, the proven benefit of transforaminal injections in comparison to the more traditional interlaminar and caudal approaches has been inconsistent. Although the evidence for transforaminal injections in treating radiculitis secondary to disc herniation and lumbar stenosis is strong [5,6], evidence is limited regarding its effect on axial pain and in patients with failed back surgery syndrome (FBSS) [5,6]. This is an important area of consideration as we anticipate a growing subset of patients with FBSS as the number of spine surgeries continues to increase.

Performing epidural steroid injections can be difficult in post-surgical patients secondary to post-operative fibrosis in the epidural space. There has been heavy reliance on caudal epidural injections for post-surgical patients due to their relative ease [[4], [5], [6], [7]]. However, this approach is not free from limitations, such as the inability to spread the injected medication to lumbar levels above L3 [8], lack of specificity for individual spinal levels, and the requirement of large injectate volumes [4]. As a result of this lack of specificity, caudal epidural injections do not provide any prognostic input when determining the contribution of a specific spinal level to a patient's symptoms, and thus have a limited role in aiding decisions regarding future surgical interventions [7,8]. In comparison, the transforaminal epidural approach efficiently instills the injectate into the epidural space and may offer prognostic input for future surgery due to its increased specificity [9,10]. One study has also demonstrated superiority of the unilateral transforaminal over the caudal injection approach in patients with FBSS [11]. However, unilateral TFESIs do not effectively cross the midline and their role is limited to relieving unilateral axial and radicular symptoms [9].

Simultaneous bilateral transforaminal epidural steroid injections have emerged as viable treatment options for patients with bilateral radicular back pain supported by three studies in the literature to date [10,12,13]. To our knowledge, there has not been any previous study performed on the efficacy of bilateral transforaminal epidural steroid injections in patients with prior lumbar spine surgery. The aim of our study is to assess the therapeutic and prognostic potential of bilateral lumbar transforaminal epidural steroid injections in patients with prior lumbar laminectomy and/or fusion.

## 2 Materials and methods

This was a retrospective case series conducted at a single academic institution's Comprehensive Spine Center. We received IRB approval prior to beginning this study. All included patients were referred for a physiatry consult for chronic back pain and radiculopathy, and evaluated between July 2017 and March 2021. Inclusion criteria were: patients who underwent bilateral transforaminal epidural steroid injections for bilateral radicular back pain, had previous laminectomy and/or fusion surgery at the symptomatic level, and had a minimum of two weeks of follow-up. Patients who had bilateral S1 TFESIs were excluded as these cannot be performed in all patients with posterior fusions, and are performed differently compared to bilateral TFESIs performed at/above the L5 levels.

Each patient was asked to describe their cumulative pain, including axial and bilateral radicular symptoms, using a numerical rating score (NRS) from 0 to 10 prior to the procedure, specifying that a score of 0 correlated with no pain and 10 correlated with excruciating pain.

**Fig. 1:**
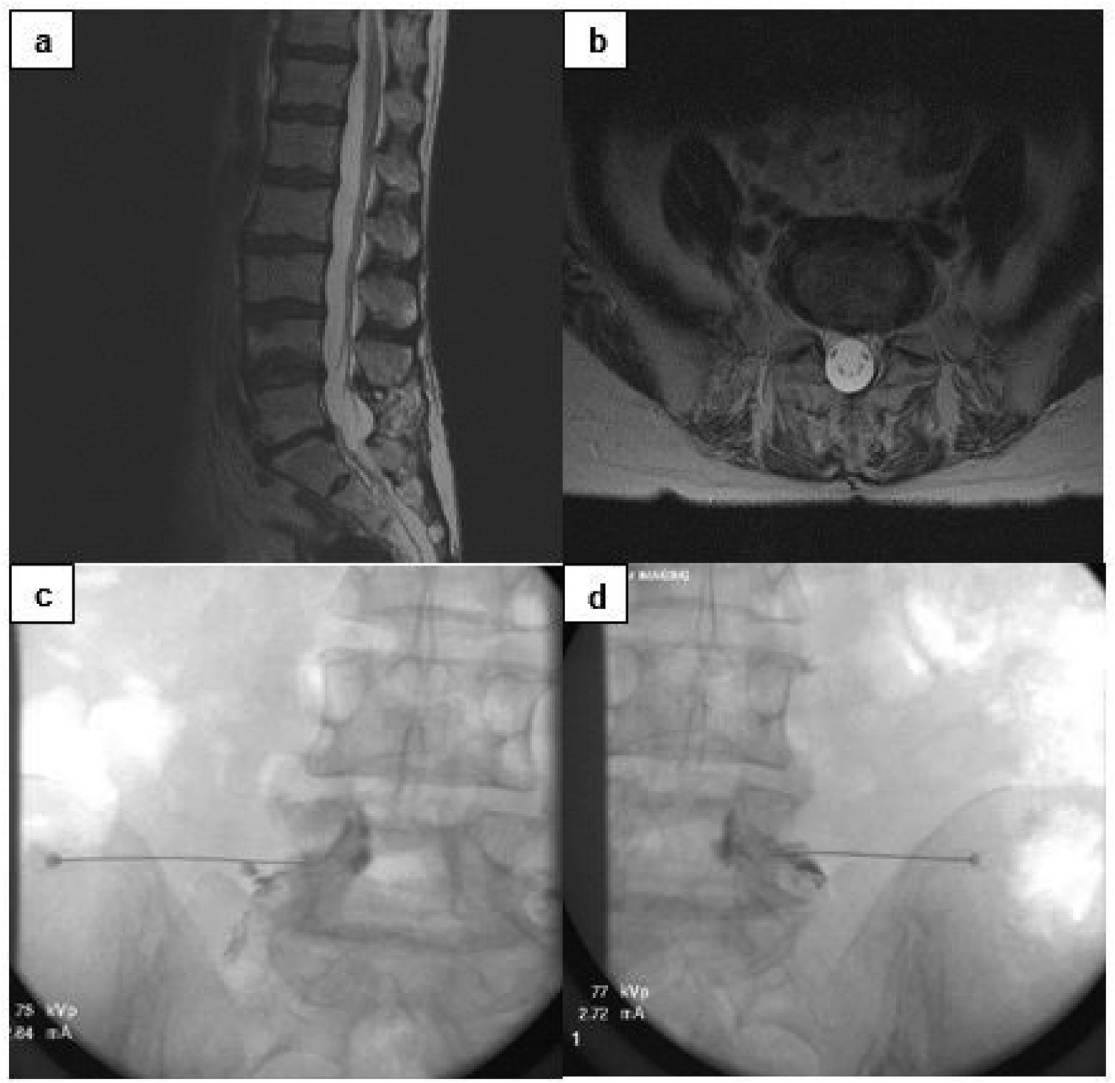
a) Sagittal T2-weighted MRI in a patient with previous laminectomy at L5-S1 **b)** Axial T2-weighted MRI at L5-S1 in same patient **c)** Fluoroscopic image showing Transforaminal epidural steroid injection at left L5-S1 **d)** Fluoroscopic image showing Transforaminal epidural steroid injection at right L5-S1 in same patient.

**Fig. 2:**
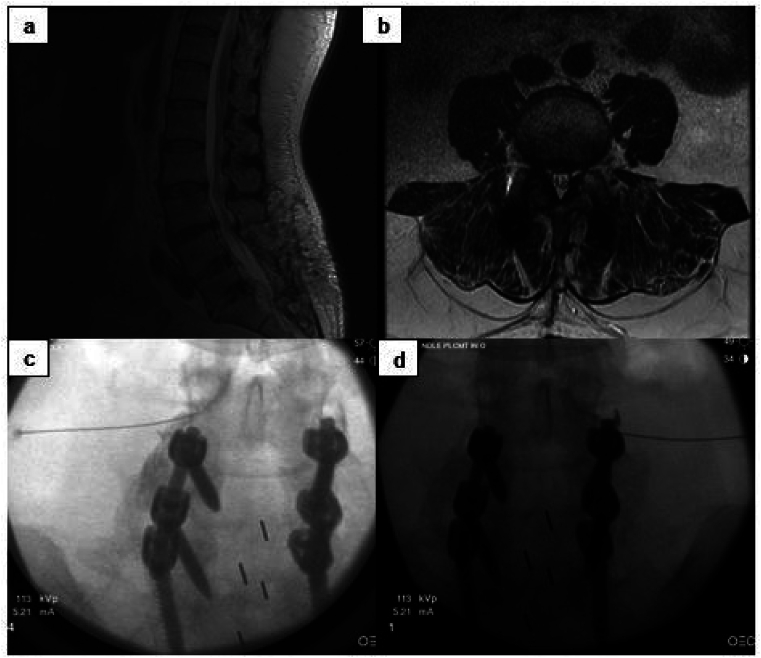
a) Sagittal T2-weighted MRI in patient with previous laminectomy and fusion **b)** Axial T2-weighted MRI at L3-4 in same patient **c)** Transforaminal epidural steroid injection at left L3-4 **d)** Transforaminal epidural steroid injection at right L3-4 in same patient.

**Fig. 3:**
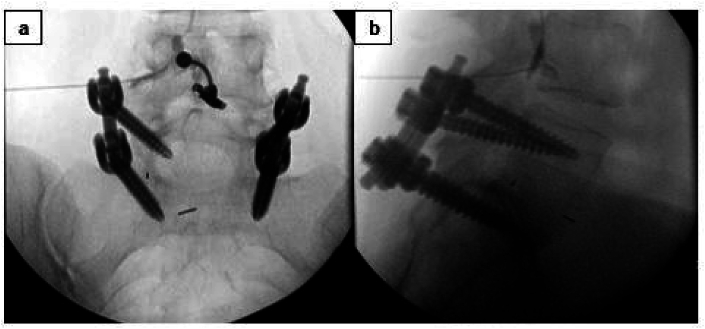
a) AP fluoroscopic radiograph depicting the epidural flow of contrast in TFESI prior to administration of injectate in same fashion b) Lateral view of same procedure depicting flow of contrast in the anterior epidural space.

The level of injection was determined based on the dermatomal pattern of radicular pain and imaging findings corresponding to the symptoms. The fluoroscopically-guided transforaminal injections were then performed following the technique outlined by Derby, Bogduk, and Kine (1993) [14]. Injections performed at levels L1-L2 and L2-L3 were administered 1 ​mL of Dexamethasone (4 ​mg/mL) combined with 1 ​mL of Lidocaine bilaterally. Injections performed at levels below L3 were administered 1 ​mL of Betamethasone (6 ​mg/mL) or 1 ​mL of Triamcinolone (40 ​mg/mL) combined with 1 ​mL of Lidocaine bilaterally. Each of the injections were reviewed for appropriate contrast spread prior to steroid injection while under fluoroscopy (Fig. 1) (Fig. 2) (Fig. 3).

Following a minimum two-week period, patients were asked for their post-injection NRS. Primary outcome measurements were: changes in NRS pre- and post-injections, if patients underwent repeat TFESIs afterwards, and whether they underwent subsequent spine surgeries involving the level of injections. Responders were defined as patients who experienced an NRS pain reduction of any degree post-injection, and non-responders as patients who experienced no change (NRS Δ ​= ​0). A minimum clinically important difference (MCID) of ≥2.0 was selected for the change in NRS to further determine the proportion of responders who experienced a clinically significant reduction of pain. Suzuki et al. (2020) previously defined the value of MCID ≥2.0 as a definitive indicator of therapeutic outcome for the change in NRS in treating patients with lumbar back pain [15].

Data analysis was performed utilizing STATA software. Statistical analysis for change in NRS was performed using a paired *t*-test with a p-value of <0.05 considered significant. Results for proportions are reported as percentages and nonparametric tests were applied when the data obtained was not normally distributed.

## 3 Results

During the study period, 464 bilateral TFESIs were performed in the lumbar region above the S1 level and three patients were excluded for receiving bilateral S1 injections. Of these, 24 patients were identified as having previous laminectomy and/or fusion surgeries. One patient did not meet inclusion criteria of reporting NRS outcomes at minimum 2 weeks post-injection. A total of 23 patients who underwent bilateral TFESI for bilateral radicular back pain following prior laminectomy and/or fusion surgery were included in this study. Each of these patients presented with bilateral radicular back pain symptoms. The mean age of the group was 61.3 ​± ​9.2 years, and 12 patients were female (52.2%). The mean length of time between previous lumbar surgery and first, or only, set of bilateral TFESIs was 9.3 years (r: 1.0–36.0 years). Fifteen patients (65.2%) had undergone prior laminectomy and fusion surgery at the level of their bilateral TFESI and 8 patients (34.8%) had undergone laminectomy alone. All patients were noted to have foraminal and/or central stenosis at the adjacent level of their prior fusion or residual foraminal stenosis at the level of their prior laminectomy. Specifically, each of the 15 patients who had previous spinal fusions had evidence of central and foraminal stenosis at the levels adjacent to their previous fusion, with minimal stenosis noted at the fusion levels themselves. Each of the 8 patients with previous laminectomy only had residual foraminal stenosis at the same level of laminectomy. The majority of patients underwent bilateral TFESIs at levels L5-S1 (34.8%), L4-L5 (30.4%), and L3-L4 (26.1%). There were no complications noted during or after any of the procedures (Table 1).

**Table 1:** **Demographics**.

Demographic	n (%)
Patients, n	23
Age	61.3 ​± ​9.2
**Gender**	
Male	11 (47.8)
Female	12 (52.2)
**Prior Back Surgery**	
Laminectomy	8 (34.8)
Laminectomy ​+ ​Fusion	15 (65.2)
**Levels of Injection**	
L1-L2	1 (4.4)
L2-L3	1 (4.4)
L3-L4	6 (26.1)
L4-L5	7 (30.4)
L5-S1	8 (34.8)

The average follow-up time was 3.7 (r: 2.0–12.0) weeks post-procedure. There were no changes in pain medications for any patients pre- and post-procedure. Four patients were on opioids prescribed by an outside provider during the study but the doses were not changed within one month prior to the TFESIs or prior to follow-up.

The average NRS pre-procedure was 7.1 out of 10 and 4.9 out of 10 post-procedure. The average reduction in NRS was 2.2, with the lower limit of pain reduction being 2 and the higher limit being 2.4 (P ​< ​0.0001) (Table 2). Overall, the cohort experienced approximately 31% absolute pain reduction. Of the 23 total patients, 16 patients (70.0%, CI 48.9–84.6%) reported any degree of NRS pain reduction post-procedure and were labeled as responders. Of the 16 responders, the absolute pain reduction was 48.8% (r: 13.3–80%). Thirteen patients experienced ≥20% pain reduction (81.3%, CI 56.2–94.2%), and 8 patients experienced ≥50% pain reduction (50.0%, CI 28.0–72.0%). Five of the responders were post-laminectomy patients, with an average NRS pain reduction of 3.5 and overall 30.9% pain reduction. The remaining 11 responders were post-laminectomy and fusion patients, achieving an average NRS pain reduction of 3.0 and overall 30.7% pain reduction.

**Table 2:** Average pre-injection, post-injection, and difference in NRS scores.

Pre-Injection	Post-Injection	Difference	P-value
7.1 ​± ​0.6	4.9 ​± ​2.2	2.2 ​± ​2.0	<0.0001∗

∗ **P-value is statistically significant**.

With the MCID defined as a NRS pain reduction ≥2, 13 of 16 responders (56.5%; CI 36.8–74.4%) achieved a clinically significant reduction in pain. Each of the 5 responders who were post-laminectomy patients met MCID criteria. Eight of the 11 responders who were post-laminectomy and fusion patients met MCID criteria.

**Fig. 4:**
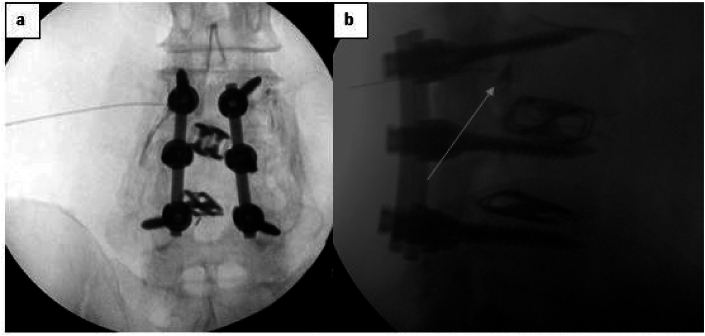
a) AP fluoroscopic radiograph depicting the radicular epidural flow of contrast in TFESI at level of previous laminectomy and fusion b) Lateral view of same procedure with arrow pointing at contrast flowing in the anterior epidural space.

Each of the bilateral TFESIs in the post-laminectomy and fusion patients were performed adjacent to the level of their fusion, except for two that were performed at the same level. These two individuals had radicular pain corresponding to the nerve roots at their fusion levels, secondary to lateral recess stenosis at the level above the fusion. There were no significant foraminal stenosis at the fusion levels. One of the patients had instrumented fusion without intertransverse fusion, so performing the injections was not significantly more challenging. The other had extensive intertransverse fusion mass and required a more lateral approach, with deeper and longer penetration through the posterior musculature, to achieve optimal intra-foraminal insertion of the needle tip. Although anterior epidural spread was noted, the classic radicular pattern of contrast was not as clearly depicted, possibly secondary to epidural scarring (Fig. 4). Regardless, both of these individuals responded favorably to the injections and achieved an average NRS pain reduction of 2.5.

Nine of the 23 patients (39.1%) underwent repeat bilateral TFESI injections, averaging 7.3 months (r: 0.9–36.0 months) after their initial injections. Of these patients, 3 had prior sole laminectomy surgery and 6 had previous laminectomy and fusion surgery, and each reported NRS pain reduction after their initial set of injections. Six underwent bilateral TFESIs at the same level as previous, 2 underwent bilateral TFESIs at an adjacent level, and 1 patient underwent a unilateral TFESI at an adjacent level. One patient did not follow-up after their second set of injections. Each of the remaining 8 patients reported an NRS pain reduction at follow-up, with an average NRS reduction of 2.2, mirroring the results of our initial set of injections. Six of the eight patients achieved MCID.

Nine of the 23 patients (39.1%) underwent further back surgery involving the same level of injections, with the operations averaging 6.5 months (r: 2–20.0 months) post-injections. This subset included 3 patients with previous laminectomy surgery and 6 patients with previous adjacent-level laminectomy and fusion surgery (Table 3). One patient underwent repeat injections 11.0 months after her first bilateral TFESIs, then subsequently underwent further back surgery 3.9 months after her second set of injections. Of the 9 patients who underwent further back surgery, 5 responded to their previous bilateral TFESIs and each reported improvements in pain and function post-operatively as well (PPV ​= ​100%). The remaining 4 patients who were non-responders to their previous bilateral TFESIs had mixed results post-operatively, with 2 reporting favorable outcomes and the other 2 reporting no change or worsening pain and function (NPV ​= ​50%) (Table 4).

**Table 3:** Comparison of number of patients between surgical groups undergoing future repeat injections and surgeries.

	Repeat Bilateral TFESI	Further Surgery
Total	9 (39.1%)	9 (39.1%)
Laminectomy	3 (13.0%)	3 (13.0%)
Laminectomy ​+ ​Fusion	6 (26.1%)	6 (26.1%)

**Table 4:** Calculating the predictive value of bilateral TFESIs in patient responses to further surgery at the same level of the injections.

	Positive Response to Further Surgery	No Response to Further Surgery	Predictive Value of Bilateral TFESIs
**Positive Response to Bilateral TFESIs**	5	0	PPV ​= ​100%
**No Response to Bilateral TFESIs**	2	2	NPV ​= ​50%

## 4 Discussion

Failed back surgery syndrome (FBSS) describes the postsurgical patient population with suboptimal outcomes, such as persistent pain and/or impaired function. The exact etiology of FBSS remains unknown but its incidence has been reported as high as 80,000 cases per year [16]. Proposed mechanisms for developing FBSS include central or foraminal stenosis adjacent to, or within, the operative level, and the degeneration of adjacent spinal levels post-laminectomy and fusion surgeries has been well-documented [[17], [18], [19], [20]]. Waguespack et al. (2002) found that foraminal stenosis was the most common cause of symptoms in their cohort of patients with FBSS, which is useful in tailoring appropriate treatment options [17].

Options for procedural management of chronic pain associated with FBSS include caudal epidural steroid injections, epidural adhesiolysis, and spinal cord stimulation [16]. Previous studies have found that epidural steroid injections can provide short-term pain relief [21], with varying successful outcomes reported. However, performing epidural steroid injections in patients with prior spinal surgery remains challenging secondary to various factors, including fibrosis of the epidural space, posterior instrumentation, and fusion mass, which can all block safe access [18], therefore limiting the role of interlaminar epidural injections as therapeutic options in patients with FBSS. Although caudal injections have been preferred due to their relative ease [7], they lack the specificity and ability to target any spinal level compared to transforaminal injections [7,8,11]. Furthermore, 2 of our patients successfully received bilateral TFESIs within the level of their previous lumbar fusions. This signifies that, albeit more challenging, performing satisfactory injections within the level of previous fusions is possible. This has not been previously reported with caudal and interlaminar injections.

Our retrospective case series affirms that simultaneous bilateral TFESIs can provide therapeutic pain relief in postsurgical patients. Minimum clinically important difference (MCID) has been used to assess therapeutic outcomes via change in NRS score after any intervention. There has been no consensus on which MCID value represents a clinically significant pain reduction, and scores have ranged from 1.7 to 2.4 in the literature [15,21,22]. Suzuki et al. (2020) most recently published a study defining MCID for NRS as a mean reduction of 2.0 post-intervention in lower back pain [21]. We selected an MCID ≥2.0 as clinically significant and the average NRS pain reduction in our study was 2.2 ​± ​0.2. Eight initial responders underwent repeat TFESIs and met follow-up criteria, and each of them responded to the second injections as well, with an average NRS pain reduction of 2.2. This signifies that repeat TFESI injections may produce a similar degree of pain reduction as the initial set.

Our results also suggest a prognostic role of bilateral TFESIs to indicate which patients would benefit from future back surgery, as each of the 5 patients who responded to their bilateral TFESIs achieved improvements in pain and function after their subsequent surgery (PPV ​= ​100%). The 4 patients who did not respond to their bilateral TFESIs had mixed results after their subsequent surgeries, which 2 reporting improved outcomes and the other 2 reporting no change or worsening outcomes (NPV ​= ​50%). To our knowledge, there are only 3 reports on the utility of bilateral TFESIs treating radicular back pain in the literature, and none of them involve post-operative lumbar surgery patients [10,12,13]. This is the first study to suggest the therapeutic and prognostic role of simultaneous bilateral TFESIs in patients with FBSS.

Our results support those obtained by Huang (2006), whose study found that TFESIs provided better pain relief compared to caudal injections in patients with FBSS [11]. Furthermore, our results also demonstrated a greater improvement in pain at and above the L3/L4 levels, an area of distribution unreachable via caudal injections. Huang suggested that the dorsal medial epidural septum may confine the spread of dorsal epidural flow to the side ipsilateral to the transforaminal and caudal injections. This can make the ability to reach adequate corticosteroid concentrations to achieve significant and sustained pain relief difficult, particularly in patients with bilateral radicular back pain. However, the ability of transforaminal injections to deliver a high concentration of injectate directly at the target combats this [11]. We suggest that performing bilateral TFESIs allows bilateral distribution of corticosteroids and perhaps even greater improvement in bilateral radicular back pain because of this.

### 4.1 Study limitations

This study has several inherent shortcomings. First, the study is limited by small sample size as a result of the referral patterns and our single interventionalist study design. Additionally, we could not control for various patient demographic factors, such as concomitant medical comorbidities, due to the retrospective and observational nature of this study. We did not include functional measures, such as the Oswestry Disability Index in assessing response to injections, which would have been beneficial in understanding baseline limitations and the effects of bilateral TSEFIs on function. Furthermore, we had a relatively short and inconsistent follow-up time, averaging 3.7 weeks (r: 2.0–12.0 weeks). This could impact results as patients who returned on the later and may have actually experienced great improvements in pain than disclosed during the follow-up visit. However, the majority of patients who did not follow-up until 10–12 weeks stated they had no recurrence of pain in the meantime. Lastly, this technique will ultimately need to be compared to prior standard of care for this patient population, which is the caudal epidural steroid injection. Despite these limitations, given the paucity of literature on this subject, the data presented here will likely be useful to practioners treating a very difficult patient population and may inspire further investigation into this effective technique.

## 5 Conclusion

This study suggests that simultaneous bilateral TFESIs have a therapeutic and prognostic role in managing patients with bilateral radicular back pain after previous lumbar spine surgery. Its procedural specificity may make it a preferable treatment compared to caudal injections, especially for patients with primarily radicular pain complaints corresponding to a dermatomal pattern consistent with stenosis on imaging. This may also help identify patients who are more likely to benefit from further surgical intervention at the level of the injections. Our findings warrant further investigation into this technique, specifically with a prospective study directly comparing bilateral TFESIs to caudal injections in post-surgical patients.

## Declarations

### Funding

No funding was received for this study.

### Availability of data and material

The datasets generated during and/or analyzed during the current study are available from the corresponding author on reasonable request.

### Ethics approval

Received IRB approval through UConn Health Center prior to start of study.

### Consent to participate-

Not applicable due to exempt status and retrospective nature of study.

## Declaration of competing interest

There were no conflicts of interest in this study.
